# Police seizure of drugs without arrest among people who use drugs in Vancouver, Canada, before provincial ‘decriminalization’ of simple possession: a cohort study

**DOI:** 10.1186/s12954-023-00833-7

**Published:** 2023-08-30

**Authors:** Kanna Hayashi, Tyson Singh Kelsall, Caitlin Shane, Zishan Cui, Vancouver Area Network of Drug Users, M.-J. Milloy, Kora DeBeck, Thomas Kerr

**Affiliations:** 1 British Columbia Centre On Substance Use, 400-1045 Howe Street, Vancouver, BC V6Z 2A9 Canada; 2 Faculty of Health Sciences, Simon Fraser University, 8888 University Drive, Burnaby, BC V5A 1S6 Canada; 3 Pivot Legal Society, 121 Heatley Avenue, Vancouver, BC V6A 3E9 Canada; 4 School of Population and Public Health, University of British Columbia, 2206 East Mall, Vancouver, BC V6T 1Z3 Canada; 5 Vancouver Area Network of Drug Users, 380 East Hastings Street, Vancouver, BC V6A 1R1 Canada; 6 Department of Medicine, University of British Columbia, 2775 Laurel Street, 10th Floor, Vancouver, BC V5Z 1M9 Canada; 7 School of Public Policy, Simon Fraser University, 515 West Hastings Street, Office 3269, Vancouver, BC V6B 5K3 Canada

**Keywords:** Harm reduction, Police, Drug laws, Overdose, Substance abuse

## Abstract

**Background:**

Several jurisdictions in Canada have recently considered decriminalizing possession of illicit drugs for personal use (henceforth, simple possession) as part of their responses to the ongoing drug toxicity/overdose crisis. In this context, we sought to examine an early implementation case of a de facto depenalization policy of simple possession offences in Vancouver, Canada, that was enacted in 2006. Specifically, we characterized experiences of people who use drugs (PWUD) whose drugs were discretionally seized by police without arrest.

**Methods:**

Data were derived from three prospective cohorts of community-recruited PWUD in Vancouver over 16 months in 2019–2021. We conducted multivariable generalized estimating equations analyses to determine the prevalence of and factors associated with drug seizure. Sub-analyses used data collected in 2009–2012 and examined the trends over time.

**Results:**

Among 995 participants who were interviewed in 2019–2021, 63 (6.3%) had their drugs seized by police at least once in the past 6 months. In multivariable analyses, factors significantly associated with drug seizure included: homelessness (adjusted odds ratio [AOR]: 1.98; 95% confidence interval [CI] 1.09–3.61), working in the unregulated drug market (AOR: 4.93; 95% CI 2.87–8.49), and naloxone administration (AOR: 2.15; 95% CI 1.23–3.76). In 2009–2012, 67.8% reported having obtained new drugs immediately after having their drugs seized by police. Odds of drug seizure were not significantly different between the two time periods (2019–2021 vs. 2009–2012) (AOR: 0.93; 95% CI: 0.64–1.35).

**Conclusions:**

Despite the depenalization policy, the Vancouver Police Department has continued to seize illicit drugs from PWUD, even in cases where no arrest occurred. This policing practice may create health and safety risks for PWUD as it forces PWUD to increase the engagement with the unregulated illicit drug market. Our findings support calls for abolishing this often-undocumented discretionary policing practice that may exacerbate ongoing health inequities and interfere with peer-based overdose prevention efforts.

## Background

Over the past two decades, growing recognition of the negative consequences of punitive, prohibition-based drug policies has led to increased support for alternative approaches worldwide [1, 2]. In this context, commonly discussed approaches include both *de jure* and de facto initiatives aimed at reducing criminal sanctions for the possession of illicit drugs for personal use (henceforth, simple possession). These initiatives can take various forms and produce different outcomes across different settings [3, 4]. According to Stevens et al. [3], decriminalization involves the *de jure* removal of criminal sanctions for simple possession, while depenalization is a de facto (non-legislative) approach intended to reduce the use of existing criminal sanctions for simple possession. Police policies instructing officers to not arrest people for simple possession are an example of depenalization. In recent years, several jurisdictions in Canada have considered ‘decriminalizing’ simple possession via a temporary legal exemption under the federal drug law (i.e. in-between *de jure* and de facto initiatives) as part of their responses to the ongoing drug toxicity/overdose crisis.

In 2006, the Vancouver Police Department (VPD), the police force within the city of Vancouver in British Columbia (BC), Canada, formalized its drug policy and endorsed harm reduction as a core pillar of its strategy, alongside prevention, treatment, and law enforcement [5]. The policy encouraged the de facto depenalization of simple possession by restricting enforcement to circumstances where people are engaged in public drug use or other behaviour that the VPD believed may harm others [5], which would notably sustain roles for policing in the lives of PWUD. Similarly, in August 2020, the Public Prosecution Service of Canada released guidelines that direct prosecutors to limit the criminal prosecution of simple possession offences to the most serious manifestations of the offence (e.g. where there is a safety risk to others) [6]. Although the VPD’s published data are limited, available data indeed indicate low and declining levels of enforcement between 2016 and 2019, with recommended charges for simple possession having decreased by 67% from 109 to 36 cases [7].

Despite VPD’s depenalization policy regarding simple possession, officers are still afforded broad enforcement discretion, including with respect to drug possession [5]. For example officers may use their ‘professional judgement’ to enforce drug seizures with or without making an arrest [5]. While anecdotal reports suggest that the police practice of drug seizure is commonplace and a driver of harm among people who use drugs (PWUD) [8], such discretionary practice is not fully captured in the VPD’s published data [9], limiting our understanding of how VPD’s policy of depenalization has been implemented at the street level.

To date, one study has quantified the extent of the discretionary police seizure of drugs without arrest in the Greater Vancouver region (mostly within the VPD jurisdiction but also including an area beyond that), showing that 9% of 465 people who injected drugs had their drugs seized by the police without arrest in the past 6 months in 2005 [10]. However, that study predated the launch of VPD’s current drug policy. In the wake of recent policy efforts towards ‘decriminalization’ in Canada and also given that drug seizures by police can greatly affect drug acquisition behaviour among PWUD and subsequently their risk of overdose and other drug-related harm [11], we sought to characterize the prevalence and associated factors of experiencing police seizure of drugs without arrest among people who used drugs at least on a daily basis (i.e. a particularly high-risk population) in Vancouver between 2019 and 2021, a period coinciding with the ongoing drug poisoning crisis [12]. We also used data collected between 2009 and 2012 to explore historical trends as well as to examine PWUD’s behaviour immediately following the drug seizures by police because this particular set of behavioural data was not collected in 2019–2021.

## Methods

### Study setting, design, and participants

Data were derived from three ongoing prospective cohort studies of PWUD in Vancouver: the Vancouver Injection Drug Users Study (VIDUS), the AIDS Care Cohort to evaluate Exposure to Survival Services (ACCESS), and the At-Risk Youth Study (ARYS). Detailed descriptions of these cohorts have been previously published elsewhere [13, 14]. In brief, VIDUS enrols HIV-seronegative adults (≥ 18 years of age) who injected drugs in the month before enrolment. ACCESS enrols HIV-seropositive adults, and ARYS enrols street-involved youth aged 14–26 who used unregulated drugs in the month before enrolment. Other common eligibility criteria across the cohorts include residing in the Greater Vancouver region and providing written informed consent. All cohorts recruit participants through word-of-mouth and street outreach primarily in two neighbourhoods in Vancouver: Downtown Eastside (DTES; an area characterized by high prevalence of marginalization and unregulated drug use) for VIDUS and ACCESS and Downtown South for ARYS. The studies use harmonized data collection procedures to allow for pooled analyses. Participants receive a CAD $40 honorarium upon completion of each study visit.

At baseline and semi-annually thereafter, participants complete an interviewer-administered questionnaire, which elicits a range of information including demographic data, substance use, health care access, and experiences with law enforcement. On 16 March 2020, data collection was suspended due to the COVID-19 pandemic on the orders of our host institutions. We resumed remote follow-up interviews (via phone) that included questions about drug seizure by police in June 2021. Options for in-person follow-up interviews were resumed in March 2022. All cohorts have received approvals from the University of British Columbia/Providence Health Care Research Ethics Board.

The cohort study survey is updated every follow-up. Survey questions related to drug seizure were only included on the questionnaire during the following periods: June 2009–November 2012; June 2019–March 2020; and June–November 2021. For the primary analysis, sample eligibility criteria included: completing a study interview at least once between June 2019 and November 2021, and reporting having resided within the city of Vancouver (i.e. within the VPD jurisdiction) and used drugs at least daily in the past 6 months. For the secondary analysis, the follow-up period from June 2009 to November 2012 was added to the eligibility while the other eligibility criteria were kept constant.

### Measures

The primary outcome was self-reported drug seizure by police without arrest in the past 6 months (yes vs. no; henceforth, drug seizure), which was derived from a question: ‘In the last 6 months, did the police take away your drugs without arresting or pressing charges against you?’ This question was asked to participants who answered yes to a previous question: ‘In the last 6 months, have you had direct contact with the police?’ Participants who reported having their drugs seized were also asked how many times they had their drugs seized within the same 6-month period. Between 2009 and 2012, another sub-question about what they did immediately afterwards was also asked. Between 2019 and 2021, participants were not asked about their immediate responses to police seizure of drugs. However, they were asked a different sub-question about which neighbourhood they were in when drugs were seized. The cases that were reported to have occurred outside the VPD jurisdiction were excluded from the analyses.

The selection of explanatory variables that we hypothesized to be associated with drug seizures by police was informed by the lived and observational experiences of the authors and the previous literature about drug seizures by police in our study setting [5, 10, 11, 15]. Additionally, the risk environment framework that has been used in the previous research characterizing PWUD–police interactions [16–20] prompted us to consider a range of social, structural, and environmental factors that may shape people’s experiences with drug seizures by police. Demographic variables included: age (continuous, per year increase); self-identified gender (man *vs.* woman, transgender or other); and self-identified ethnicity/ancestry (white *vs.* Indigenous or other persons of colour). Substance use-related variables referring to the past 6 months included: ≥ daily use of unregulated opioids (i.e. heroin, fentanyl, or *down* [a colloquial term locally used to refer to unregulated opioids]), unregulated stimulants (i.e. crystal methamphetamine or powder/crack cocaine), or cannabis, respectively; self-reported experience of opioid withdrawal, defined by answering yes to a question: ‘In the last 6 months, have you gone through opioid withdrawal (gotten dopesick)?’; always or usually (i.e. at least 75% of the time) injected drugs in public places (yes vs. < 75% of the time or not injected drugs); non-fatal overdose; and having administered naloxone to someone for overdose reversal. Social and structural exposures in the past 6 months included: homeless, sex work, working in unregulated drug markets (e.g. direct selling, middling, steering, holding, enforcing, cooking/packaging/producing, and supplying as defined in a previous study [21]), street-based income generation activities (e.g. recycling, panhandling, etc.), incarceration, and self-reported inability to access health and/or social services. The calendar year of interviews (2021 vs. 2019–2020) was also included to account for potential differences before and during the COVID-19 pandemic. Unless otherwise stated, all variables were dichotomized as yes vs. no. Missing responses were less than 3% (maximum 2.1%) per variable and were treated as missing in variable categorization and statistical analyses.

### Primary analysis: factors associated with drug seizure by police in 2019–2021

We first examined the sample characteristics stratified by ever-reporting drug seizure during the study period, using the Wilcoxon rank-sum test (for age) and the Pearson’s Chi-squared test (for all other variables). Next, to account for the repeated measures, we used generalized estimating equations (GEE) with logit link to examine associations with drug seizure. We built a multivariable model by using an a priori-defined backward model selection procedure based on examination of Akaike Information Criterion and including all variables that were associated with the outcome at *p* < 0.10 in bivariable analyses in the initial full model [22]. The multicollinearity was assessed by checking the variance inflation factor with a cutoff of 5.0 [23].

### Secondary analysis: trends of drug seizure between 2009–2012 and 2019–2021

We sought to examine whether the annual prevalence of drug seizure was significantly different between the two time periods (2009–2012 vs. 2019–2021) after accounting for potential differences in the sample characteristics. As a first step, we employed GEE with logit link to compare the characteristics of the participants in the two periods. We used all the variables used for the primary analysis as covariates, except for naloxone administration and opioid withdrawal because the questions for these two variables were not consistently asked during the 2009–2012 period. The calendar year of interviews used for the primary analysis (2021 vs. 2019–2020) was replaced with 2009–2012 vs. 2019–2021. We also added cohort designation (VIDUS vs. ACCESS vs. ARYS) as a covariate to account for potential differences across cohorts. Next, we included all variables that were associated with the variable comparing the two periods at *p* < 0.05 in bivariable analyses as covariates in a multivariable GEE model where drug seizure was the dependent variable, and the variable comparing the two periods was the primary independent variable. All *p*-values were two-sided. All statistical analyses were performed using SAS 9.4 (Cary, North Carolina, USA).

## Results

### Factors associated with drug seizure during 2019–2021

A total of 995 participants were eligible for the primary analysis and contributed 1696 observations. Between 2019 and 2021, 63 (6.3%) participants had at least one report of drugs being seized, with a total of 68 reports (35 reports in 2019, 10 in 2020, and 23 in 2021). Of the 68 reports, 55 (80.9%) mentioned that it occurred in the DTES neighbourhood. Further, 42 (61.8%) reports also included the number of occurrences in the past 6 months, with 23 (54.8%) reporting once, 11 (26.2%) twice, and 8 (19.0%) more than twice. Table 1 shows some sample characteristics at their most recent study visit. As shown, the median age was 41 (1st and 3rd quartiles: 31 and 54) years, 588 (59.1%) self-identified as a man, and 540 (55.1%) self-identified as white, 382 (38.9%) as Indigenous, and 59 (6.0%) as other persons of colour. The prevalence of experiencing non-fatal overdose in the past 6 months was significantly higher among those who reported having their drugs seized compared to those who did not (30.2% vs. 18.3%, *p* = 0.020) as was the prevalence of experiencing opioid withdrawal in the past 6 months (71.4% vs. 48.8%, *p* = 0.001).

**Table 1:** Most recent characteristics of VIDUS/ACCESS/ARYS participants who reported at least daily use of drugs in the past 6 months in Vancouver, Canada, 2019–2021 (*n* = 995)

Characteristic	Total *n* (%) 995 (100%)	Reporting police seizure of drugs without arrest^a^ during the study period		*P*-value
		Yes *n* (%) 63 (6.3%)	No *n* (%) 932 (93.7%)	
Cohort designation				
VIDUS	437 (43.9%)	35 (55.6%)	402 (43.1%)	0.105
ACCESS	271 (27.2%)	11 (17.5%)	260 (27.9%)	
ARYS	287 (28.8%)	17 (27.0%)	270 (29.0%)	
Calendar year of interview				
2021	522 (52.5%)	23 (36.5%)	499 (53.5%)	0.009
2019–2020	473 (47.5%)	40 (63.5%)	433 (46.5%)	
Age (median, 1st, and 3rd quartiles)	41 (31–54)	36 (31–42)	42 (31–55)	0.001
Self-identified ethnicity/ancestry				
Indigenous	382 (38.9%)	17 (27.0%)	365 (39.8%)	0.051
Person of colour	59 (6.0%)	7 (11.1%)	52 (5.7%)	
White	540 (55.1%)	39 (61.9%)	501 (54.6%)	
Self-identified gender^a^				
Woman, transgender, other	407 (40.9%)	19 (30.2%)	388 (41.6%)	0.073
Man	588 (59.1%)	44 (69.8%)	544 (58.4%)	
≥ Daily unregulated opioids use^a,b^	580 (58.3%)	50 (79.4%)	530 (56.9%)	0.001
≥ Daily unregulated stimulants use^a,c^	543 (54.6%)	45 (71.4%)	498 (53.4%)	0.006
≥ Daily cannabis use^a^	360 (36.8%)	14 (23.0%)	346 (37.7%)	0.021
Opioid withdrawal^a^	498 (50.3%)	45 (71.4%)	453 (48.8%)	0.001
Always/usually injected drugs in public places^a^	90 (9.1%)	14 (22.6%)	76 (8.2%)	< 0.001
Non-fatal overdose^a^	189 (19.0%)	19 (30.2%)	170 (18.3%)	0.020
Administered naloxone to someone^a^	283 (29.5%)	32 (53.3%)	251 (27.9%)	< 0.001
Homeless^a^	219 (22.1%)	28 (45.2%)	191 (20.6%)	< 0.001
Sex work^a^	121 (12.3%)	11 (17.5%)	110 (11.9%)	0.197
Working in the unregulated drug market^a^	268 (26.9%)	40 (63.5%)	228 (24.5%)	< 0.001
Street-based income generation (e.g. recycling and panhandling)^a^	265 (26.6%)	23 (36.5%)	242 (26.0%)	0.067
Incarceration^a^	73 (7.4%)	24 (38.1%)	49 (5.3%)	< 0.001
Inability to access health and/or social services^a^	245 (24.8%)	24 (38.1%)	221 (23.9%)	0.012

ACCESS: The AIDS Care Cohort to evaluate Exposure to Survival Services Study. ARYS: The At-Risk Youth Study. VIDUS: The Vancouver Injection Drug Users Study

For the calculation of percentages, missing observations (max. 2.1% per variable) were excluded from the denominators

*P*-values were derived from the Pearson’s Chi-squared test for all variables except for age, which used the Wilcoxon rank-sum test

^a^Refers to behaviours and events in the 6 months prior to an interview

^b^Unregulated opioids include heroin, fentanyl, *down*, speedball, or goofball

^c^Unregulated stimulants include crystal methamphetamine, powder/crack cocaine, speedball, or goofball

Table 2 presents the results of bivariable and multivariable GEE analyses. Following our modelling procedures, five variables remained in the final multivariable model, and all of them remained significantly associated with having one’s drugs seized: older age (adjusted odds ratio [AOR]: 0.97; 95% confidence interval [CI] 0.94–0.99), self-identifying as a man (AOR: 1.98; 95% CI 1.10–3.56), naloxone administration (AOR: 2.15; 95% CI 1.23–3.76), homelessness (AOR: 1.98; 95% CI 1.09–3.61), and working in the unregulated drug market (AOR: 4.93; 95% CI 2.87–8.49). We did not detect any collinearity.

**Table 2:** Bivariable and multivariable generalized estimating equations analyses of factors associated with police seizure of drugs without arrest among people who used drugs daily in Vancouver, Canada, 2019–2021 (*n* = 995)

Characteristic	Odds ratio			
	Unadjusted (95% CI)	*P*-value	Adjusted (95% CI)	*P-*value
Year of interview (2021 vs. 2019–2020)	1.10 (0.67–1.79)	0.708	–	
Age (per year older)	0.97 (0.95–0.99)	< 0.001	0.97 (0.94–0.99)	0.008
Self-identified as white	1.35 (0.80–2.28)	0.267	–	
Self-identified as a man^a^	1.68 (0.97–2.91)	0.066	1.98 (1.10–3.56)	0.023
≥ Daily unregulated opioids use^a,b^	2.83 (1.51–5.30)	0.001	–	
≥ Daily unregulated stimulants use^a,c^	2.22 (1.30–3.81)	0.004	–	
≥ Daily cannabis use^a^	0.49 (0.27–0.91)	0.025	–	
Opioid withdrawal^a^	2.37 (1.37–4.11)	0.002	–	
Always/usually injected drugs in public places^a^	3.34 (1.80–6.18)	< 0.001	–	
Non-fatal overdose^a^	2.07 (1.23–3.47)	0.006	–	
Administered naloxone to someone^a^	2.89 (1.71–4.89)	< 0.001	2.15 (1.23–3.76)	0.007
Homeless^a^	3.10 (1.85–5.20)	< 0.001	1.98 (1.09–3.61)	0.026
Sex work^a^	1.60 (0.83–3.08)	0.161	–	
Working in the unregulated drug market^a^	5.86 (3.50–9.78)	< 0.001	4.93 (2.87–8.49)	< 0.001
Street-based income generation^a^	1.67 (1.02–2.74)	0.041	–	
Incarceration^a^	10.59 (6.09–18.41)	< 0.001	–	
Inability to access health and/or social services^a^	1.94 (1.14–3.32)	0.015	–	

CI: Confidence interval

^a^Refers to behaviours and events in the 6 months prior to an interview

^b^Unregulated opioids include heroin, fentanyl, *down*, speedball, or goofball

^c^Unregulated stimulants include crystal methamphetamine, powder/crack cocaine, speedball, or goofball

### Trends of drug seizure between 2009–2012 and 2019–2021

In total, 1894 VIDUS/ACCESS/ARYS participants were eligible for the secondary analysis and contributed 4607 observations. Of these individuals, 410 (21.7%) contributed at least one observation in each of the two time periods (2019–2021 and 2009–2012), 911 (48.1%) in 2009–2012 only, and 584 (30.8%) in 2019–2021 only. A total of 1266 (66.8%) participants completed more than one interview during the study period and were followed up for a median of 2.4 (1st and 3rd quartiles: 1.7 and 9.8) years. The median age at their most recent observation was 42 (1st and 3rd quartiles: 30 and 52) years, 1188 (63.2%) self-identified as a man, and 1080 (57.6%) self-identified as white, 679 (36.2%) as Indigenous, and 117 (6.2%) as other persons of colour.

Overall, 214 (11.3%) individuals reported having their drugs seized at least once with a total of 259 reports. Among the 214 participants, 149 (69.6%) were interviewed between 2009 and 2012 and answered the sub-question about what they did immediately afterwards. The most common response was acquiring more drugs (67.8%), followed by doing nothing (20.8%), got fronted drugs or money (5.4%), borrowed drugs or money (2.7%), and sold drugs (2.7%). The annual prevalence of drug seizure is shown in Table 3. An average annual prevalence was 5.6%. In the GEE analyses, the variable comparing the two time periods (2019–2021 vs. 2009–2012) was not significantly associated with drug seizure in either the unadjusted (odds ratio: 0.85; 95% CI 0.64–1.15) or adjusted analysis (AOR: 0.93; 95% CI 0.64–1.35).

**Table 3:** Annual prevalence of police seizure of drugs without arrest in the past 6 months among people who used drugs daily in Vancouver, Canada, 2009–2021

Calendar year	2009 (June–December)	2010	2011	2012 (January–November)	2019 June–2020 March	2021 (June–November)
N interviewed	711	816	773	699	858	533
N and % (95% CI) reporting drug seizure^a^	49 6.9 (5.0–8.8)	66 8.1 (6.2–10.0)	43 5.6 (3.9–7.2)	23 3.3 (2.0–4.6)	44 5.1 (3.6–6.6)	23 4.3 (2.6–6.0)

Because data were collected only for 7 months in 2019 and two and a half months in 2020, these 2 years’ data were combined when estimating the annual prevalence

CI: Confidence interval

^a^The number refers to the number of participants providing at least one report of police seizure of drugs without arrest in the past 6 months

## Discussion

During the 16-month study period between June 2019 and November 2021 (June 2019–mid-March 2020 and June 2021–November 2021), 6% of our sample of people who used drugs daily in Vancouver reported having had their drugs seized by police without arrest at least once in the past 6 months. When examining the historical trends of annual prevalence, we found a declining trend in reports of drug seizure from 7% in 2009 to 3% in 2012, while the prevalence between June 2019 and mid-March 2020 and between June and November of 2021 (4–5%) remained essentially the same as the annual prevalence in 2011–2012. However, overall, the odds of drug seizure were not significantly different between the two time periods (2019–2021 vs. 2009–2012).

The low documented numbers of recommended charges for simple possession by the VPD are often cited to indicate success of VPD’s de facto depenalization policy [24]. Certainly, recommended charges for simple possession and drug seizure without arrest are two distinct practices and not directly comparable; however, given that statistics regarding the former are almost the only data used to assess the extent of depenalization, it is worth examining the potential discrepancy between the two to deepen our understanding of street-level drug law enforcement activities. For example, in 2019, VPD recommended 36 charges for simple possession to Crown Counsel [7]. In contrast, in our study, participants reported experiencing at least 35 drug seizures by police during the 6 months prior to their interview date between June and December 2019. The number of unique events was much higher than 35 given that a substantial portion of participants (approx. 45% of those who reported the number of occurrences of police seizure of drugs) experienced having their drugs seized more than once during the same 6-month period. These findings corroborate previous anecdotal reports [8] and show that drug seizure without arrest occurs more frequently than the VPD’s recommended charges for simple possession.

Some negative consequences of criminal justice involvement may be avoided by police not recommending charges for simple possession. However, we found that more than two-thirds of PWUD who were interviewed in 2009–2012 obtained more drugs immediately after police seized their drugs. These findings suggest that this policing practice may still lead to health and safety harms for PWUD. For example a previous qualitative study that interviewed PWUD in 2017 described that police seizure of drugs inadvertently promoted the creation of drug debts and increased the risk of drug market violence among PWUD [11]. Some PWUD were also forced to refill their drug supply hastily from an unknown unregulated drug market worker especially when experiencing withdrawal [11, 25]. Each time an individual has to return to the unregulated market, especially if accessing drugs from an unknown source, they are increasing their risk of fatal or non-fatal overdose. In this regard, drug seizure essentially ‘mimics the health and safety harms associated with criminalization’ [15], undermining the intended benefits of the VPD’s depenalization policy. Of concern, a previous qualitative study reported that some police officers in BC believed that seizure of drugs is ‘beneficial for preventing harms, including overdose’, though it was not made clear whether it referred to VPD officers or other officers in BC or both [26].

When examining the data collected in 2019–2021, we also found that participants who administered naloxone to reverse someone’s overdose were more likely to have their drugs seized. As we did not directly ask our participants whether naloxone administration and police seizure of drugs occurred at the same time, future research needs to investigate this association in more depth. The VPD has a policy of non-attendance at overdose events unless requested by emergency health services [27]. However, a previous ethnographic study documented regular police presence in the DTES neighbourhood [28], which may mean that police officers are incidentally present near overdose events in public spaces. Even when police officers attend an overdose event, the *Good Samaritan Drug Overdose Act* (enacted in Canada in 2017) provides that no person who experiences or witnesses an overdose shall be charged or convicted for simple possession [29]. But if police is seizing drugs from PWUD who administered naloxone for overdose reversal at an overdose scene, the purpose of the *Good Samaritan Drug Overdose Act* is arguably undermined even in the absence of charges, because fear of police engagement at overdose events would persist among PWUD [29]. Indeed, a recent qualitative study based on interviews with PWUD in BC indicated that police seizure of drugs at overdose scenes was common, though it was not clear how common it was specifically in Vancouver [30]. Alternatively, PWUD who administered naloxone may have had their drugs seized by police at locations other than overdose scenes. Naloxone kits carried by these PWUD might ‘mark’ them as a person who uses drugs, thereby attracting police attention and resulting in drug seizures. Again, more research is needed to investigate detailed contexts around this finding.

Our results from the 2019–2021 data also demonstrated that those who were experiencing greater marginalization (e.g. homelessness and working in the unregulated drug market) were more likely to have their drugs seized. Overall, our research findings indicate that police seizure of drugs without arrest has potential to exacerbate ongoing health inequities and potentially undermine peer-based overdose prevention efforts. Therefore, the findings support calls for formally abolishing this discretionary policing practice. The efforts to achieve this goal may include Health Canada issuing a guidance about the harms of the practice to discourage police from engaging in it.

Our study results come at a crucial moment as BC will begin a trial period of ‘decriminalization’ respecting the simple possession of a cumulative quantity of 2.5 g of certain illicit drugs in January 2023, via a legal exemption under the federal drug law [31]. While in principle, the move could have public health benefits, unfortunately, feedback from PWUD communities and others indicates that the threshold quantity of drugs that defined simple possession is too low to be beneficial [32–34]. According to Health Canada, those who possess less than 2.5-g total of certain drugs for personal consumption will typically not be charged for simple possession and police will not seize those drugs [35]. Their approach to drug seizures (namely that ‘the drugs will not be seized’ if below 2.5 g [35]) was developed by BC’s Ministry of Mental Health and Addictions and Health Canada and would thus seem to suggest acknowledgement of the public health harms associated with the practice. However, it is unknown how BC’s ‘decriminalization’ model will influence drug seizures when people possess *more than* 2.5 g of drugs—or even when people possess less than that amount, given oft-noted discrepancies between policies and street-level practices [36]. Our findings suggest that it is important to evaluate the impacts of BC’s emerging policy on the police practice of seizing drugs without arrest particularly among PWUD who are socioeconomically more disadvantaged and more visible to police, including those who are homeless or working in the unregulated drug market for subsistence [21].

This study has limitations. First, a non-random sample limits the generalizability of our findings. Second, self-reported data may be influenced by some reporting bias, although reasonable validity of self-reports has been demonstrated in a similar population [37]. Third, data were not consistently collected between June 2009 and November 2021 (i.e. no data collection between December 2012 and May 2019 or between mid-March 2020 and May 2021), which made it challenging to determine the longitudinal patterns of drug seizure. Also, while we restricted the sample to those residing in the VPD jurisdiction and the cases of drug seizure between 2019 and 2021 to those reported to have occurred in the VPD jurisdiction, the outcome measure may still have included cases occurring outside the VPD jurisdiction due to potential self-report errors or missing responses to the locations of police drug seizures. Lastly, overall rates of police seizure of drugs (*with and without* arrest) would be much higher than those identified in the present study, which focused on cases *without* arrest. Notably, in relation to their drug seizures data in 2019–2020, the VPD’s Director of Public Affairs also commented that smaller drug seizures often involved an arrest for another offence [9, 38]. While the present study is limited in this regard, its unique strength is that it elucidated the extent to which police seizure of drugs occurred, even when PWUD were presumably not engaged in any serious offences that necessitated an arrest.

## Conclusions

In sum, we found that 6% of our sample of PWUD in Vancouver had their drugs seized by police without arrest over a 16-month period in 2019–2021. In 2019, police seizure of drugs without arrest appeared to have occurred more frequently than simple possession charges recommended by the VPD. Drug seizures without arrest were concentrated among socioeconomically more marginalized PWUD and those who administered naloxone to others for overdose reversal. In addition, our data collected in 2009–2012 showed that the majority of PWUD who had their drugs seized acquired new drugs immediately following said seizure. This finding indicates that the police seizure of drugs without arrest can lead to more frequent interactions with the unregulated drug market, sometimes with direct impacts on their health and safety, including but not limited to fatal overdose. These findings call for abolishing this harmful discretionary policing practice that may aggravate ongoing health inequities and interfere with peer-based overdose responses.

## Data Availability

The data used for this study are not publicly available due to the way informed consent was obtained from study participants.

## Abbreviations

ACCESS: AIDS Care Cohort to evaluate Exposure to Survival Services
AOR: Adjusted odds ratio
ARYS: At-risk youth study
BC: British Columbia
CI: Confidence interval
DTES: Downtown eastside
GEE: Generalized estimating equations
PWUD: People who use drugs
VIDUS: Vancouver Injection Drug Users Study
VPD: Vancouver Police Department
